# Retrospective study of the efficacy of vascularized tissue transfer for treating antibiotic-resistant bacteria-infected wound: Comparison with clean and antibiotic-sensitive bacteria-infected wound

**DOI:** 10.1097/MD.0000000000025907

**Published:** 2021-06-11

**Authors:** Seong Hwan Kim, Ju Ho Lee, Seong Eun Kim, Se Ho Shin, Hyeon Jo Kim, Seong Joo Lee, Jae Hyun Kim, In Suck Suh

**Affiliations:** a Department of Plastic and Reconstructive Surgery, Kangnam Sacred Heart Hospital, Hallym University College of Medicine, Seoul; b Department of Internal Medicine, Division of Infectious Diseases, Chonnam National University Hospital, Gwangju, Korea.

**Keywords:** antibacterial agents, antibiotic resistance, infection, surgical flaps, wound healing

## Abstract

If wounds are infected with bacteria resistant to an empirical antibiotic regimen, effective wound treatment will be delayed. This can delay wound healing and lengthen hospital stays, increasing the costs to patients. Long-term antibiotic use can also result in minor and major complications, such as diarrhea, antibiotic resistance, or life-threatening leukopenia. Multidrug-resistant (MDR) bacteria make wound treatment even more difficult. Traditionally, surgeons thought that adequate infection control should be established before soft tissue coverage. However, wounds infected by MDR do not heal well with this traditional method and there are no optimal treatment guidelines for MDR bacteria-contaminated wounds.

We reviewed 203 patients who underwent vascularized flap surgery from 2012 to 2019 to cover wounds. Class IV and I wounds were compared according to the Centers for Disease Control and Prevention classification. Class IV was further classified as antibiotic-resistant (ARB) and antibiotic-sensitive (ASB) bacteria. Wound size, mode, location, pathogens, healing time, and basic demographics were evaluated. Data were compared using Cramer's V and one-way ANOVA or independent *t* tests.

The average healing time was longer in the ARB (19.7 [range 7–44] days) and ASB (17.9 [range 2–36] days) groups than in the Clean group (16.5 [range 7–28] days). Healing time differed in the 3 groups (*P* = .036). It was longer in the class IV group than in the class I group (*P* = .01). However, it was not statistically different between the ARB and ASB groups (*P* = .164).

In our study the difference in healing time was small when vascularized tissue transfer was done in ARB-infected wound compared with ASB-infected and clean wound. It is necessary to perform surgery using vascularized tissue for the infected wound of antibiotic-resistant bacteria.

## 1 Introduction

The Centers for Disease Control and Prevention (CDC) has classified wound status into 4 classes. Class 1 wounds are considered clean; they are not infected, no inflammation is present, and are primarily closed. Class 2 wounds are considered to be clean-contaminated, that is, they lack unusual contamination. Bacteria from class 2 wounds can enter the respiratory, alimentary, genital, or urinary tracts. Class 3 wounds are considered to be contaminated; these are fresh, open wounds that can result from inadequate sterile techniques or leakage from the gastrointestinal tract into the wound. Class 4 wounds are considered dirty-infected. These wounds typically result from improper care of traumatic wounds. Class 4 wounds contain devitalized tissue and they are most commonly infected by microorganisms present in perforated viscera or the operative field.^[1]^

Antibiotic resistance refers to the ability of bacteria to resist the effects of antibiotics.^[2]^ If the bacteria are resistant to an empirical antibiotic regimen, effective treatment will be delayed, which can delay wound healing and lengthen hospital stays. Long-term antibiotic use can result in complications such as diarrhea, antibiotic resistance, or even life-threatening leukopenia.^[3–8]^ Infected chronic wounds are a health problem of enormous magnitude, affecting many hundreds of thousands of patients. Uncontrolled and infected wounds lead to prolonged hospitalization and increased use of medical resources and threaten optimal care. Moreover, prolonged infection may spread to other patients through the patients themselves, medical staff, or equipment and associated with hospital-acquired infections such as ventilator-associated pneumonia and catheter-associated infections.^[9]^ The healthcare cost amounts to billions of dollars annually worldwide.

Multidrug-resistant (MDR) isolates are defined as those resistant to 3 or more classes of antipseudomonal agents (i.e., penicillins/cephalosporins, carbapenems, fluoroquinolones, and aminoglycosides). Frequently isolated pathogens include methicillin-resistant *Staphylococcus aureus* (MRSA), vancomycin-resistant enterococci, *Acinetobacter baumannii*, Enterobacteriaceae that produce extended-spectrum beta-lactamases or carbapenemases, and carbapenem-resistant *Pseudomonas aeruginosa* are.^[2,10]^ The risk factors for resistant infections are older age, underlying comorbid conditions (e.g., diabetes, renal failure, malignancies, and immunosuppression), long hospital stay, and receipt of antimicrobial therapy.^[11]^

A wound is generally considered chronic if it has not healed in 4 weeks. Chronic wounds have also been defined as wounds that do not show a 20% to 40% reduction in area after 2 to 4 weeks of optimal therapy. Patients with chronic wounds tend to need longer hospitalization because they have multiple comorbidities, and the wound itself can have a negative effect on the patient's condition. As they stay longer in the hospital, the possibility of drug resistance of the bacteria colonizing the wound increases. MDR bacteria such as MRSA and carbapenem-resistant *Pseudomonas* aeruginosa can delay wound healing, while determining the appropriate antibiotics to treat the bacteria. Moreover, surgeons may hesitate to perform surgical treatment on a chronic wound colonized with MDR bacteria considering the postoperative surgical site wound infection and seromas.

Traditionally, surgeons believed that adequate infection control should be established before soft tissue coverage,^[12]^ especially in fulminant infections. Therefore, attempts at infection control and confirmation of negative wound culture results led to delays in surgical management and discharge, which was inefficient and suboptimal. However, there are no optimal treatment guidelines for MDR bacteria-contaminated wounds. Surgeons who want to prepare a wound properly for surgery may be confused about how to manage MDR-bacteria contaminated wounds. Inadequate surgical debridement and antibiotic use can delay the recovery of an MDR bacteria-contaminated wound, which can extend the defect size and depth of the wound.

In this study, we chose early intervention to treat this difficult problem with a little technical advancement. In our experience with simple treatment principles over years of managing infected wounds, these wounds can be treated without trouble. The most important principle is *en bloc* resection, which refers to the radical debridement of the infected tissue. The purpose of the study is to establish simple but useful surgical principles by comparing wound-healing times between infected wounds and clean surgical wounds when those wounds are treated with the same principles.

## 2 Methods

### 2.1 Patients

From 2012 to 2019, patients who had vascularized flap surgeries were investigated retrospectively through chart reviews. Age, sex, comorbidities, location and cause of the wound, and types of colonizing bacteria were analyzed. Patients with class IV (dirty-infected) wounds following the CDC wound classification or class I (clean) wounds, such as those from tumor ablation and nevus removal (clean surgery group), were selected.^[1]^ The patients with dirty-infected wounds were further classified into the following 2 groups based on the culture results: the antibiotic-resistant bacteria (ARB) group was infected with MDR bacteria and the antibiotic-sensitive bacteria (ASB) group was infected with bacteria that were not considered to be MDR bacteria. Patients requiring multiple surgeries or with multiple wound locations were excluded.

All patients were treated with flaps on hospitalization. All patients with class IV wounds were treated with empirical antibiotics at first. When the patients were referred to our department, infected tissue from the wound was cultured. We changed the antibiotics according to the results of the wound culture and sensitivity assessment. We usually maintained the antibiotics for a few days after suture removal. In the clean surgery group, the antibiotics were administered in the operating room right before surgery. Tables 1 and 2 show the antibiotics used and dosages. The dosages were adjusted according to patient condition (weight, liver and kidney function, etc.).

**Table 1:** The routinely used pre- and postoperative antibiotics.

	Preoperative antibiotic use (Top 3)	Postoperative antibiotics use (Top 3)
ARB group	First-generation cephalosporin	Fourth-generation cephalosporin
	Penicillin-derived antibiotics	Glycopeptide (Teicoplanin)
	Aminoglycoside	Ureidopenicillins (Piperacillin)
ASB group		Third-generation cephalosporin
		Penicillin-derived antibiotics
		First-generation cephalosporin
Clean surgery	None	First-generation cephalosporin

ARB = antibiotic-resistant bacteria, ASB = antibiotic-sensitive bacteria.

**Table 2:** The mostly used antibiotics and injection route.

Antibiotics		Dose	Route
mechanism	Name		
Penicillin-derived antibiotics	Amphicillin sodium	1g/8hr	IV
First-generation cephalosporin	Cefazolin sodium	1g/12hr	IV
Third-generation cephalosporin	Ceftriaxone sodium hydrate	1g/12hr	IV
Fourth- generation cephalosporin	Cefepime hydrochloride hydrate	1g/12hr	IV
Ureidopenicilin	Piperacillin-tazobactam	4g-0.5g/8hr	IV
Aminoglycoside	Netilmicin sulfate	0.15g/2hr	IV or IM
Glycopeptide	Vancomycin hydrochloride	1g/12hr	IV
	Teicoplanin	400mg/12hr for 36 hr	IV
		Then 400mg/24hr	
Tetracycline	Tigecycline	50mg/12hr	IV

IM = intramuscular, IV = intravenous.

### 2.2 Statistical analysis

Patient demographics, including age, sex, and comorbidities, were recorded and compared. Wound size, mode, and location were evaluated. Pathogens colonizing the wound were determined. The wound-healing time was measured from the time the patients were referred to our clinic to the time antibiotics were stopped after wound healing. In the clean surgery group, the wound-healing time was between the surgery and when the antibiotics were stopped. Nominal variables were compared using Cramer's V. Numerical variables were compared using one-way analysis of variance or the independent *t* test according to the numbers of independent variables. A *P* value < .05 was taken to be statistically significant. Statistical analyses were performed using IBM SPSS Statistics for Windows (ver. 22.0, IBM, Armonk, NY).

### 2.3 Ethics statement

This study conformed to the ethical guidelines of the 1975 Declaration of Helsinki and was reviewed and approved by the Institutional Review Board of Kangnam Sacred Heart Hospital, College of Medicine, Hallym University (IRB number 2018-06-013).

## 3 Results

### 3.1 Patient demographics

The study enrolled 203 patients: 80 in the ARB group, 72 in the ASB group, and 51 in the clean surgery (Clean) group (Table 3). Their mean age was 60 (range 11–89) years in the ARB group, 55 (range 19–87) years in the ASB group, and 57 (range 13–87) years in the Clean group. The male-to-female ratio was 41:39, 35:37, and 27:24 in the ARB, ASB, and Clean groups, respectively. Age and sex distribution did not differ significantly among the groups (*P* = .26 and *P* = 0.888, respectively)

**Table 3:** Basic patient demographics and study results.

	Group			Total	*P* value		
	ARB	ASB	Clean		ARB-ASB-clean	ARB- ASB	CLASS I–IV
Number of cases	80	72	51	203	–		
Age (mean ± SD)	60 ± 16.2	55 ± 19.2	57 ± 20.1	58 ± 19.2	0.26^∗^		
Male: female	41:39	35:37	27:24	103:100	0.888^†^		
Underlying diseases							
Hypertension	38 (47.5%)	23 (31.9%)	13 (25.45%)	74 (36.5%)			
Diabetes	31 (38.8%)	20 (27.7%)	11 (21.6%)	62 (30.5%)			
PAOD	7 (8.8%)	3 (4.2%)	1 (2%)	11 (5.4%)			
Status of infection							
Chronic	56 (70.0%)	50 (69.4%)	–	106 (69.7%)			
Acute	24 (30.0%)	22 (30.6%)	–	46 (30.3%)			

ARB = antibiotic-resistant bacteria, ASB = antibiotic-sensitive bacteria, PAOD = peripheral arterial occlusive disease, SD = standard deviation.

∗ One-way ANOVA test.

† Cramer V test.

### 3.2 Wound characteristics

Chronic wounds including pressure sores were most common in both the ARB and ASB groups, whereas tumor ablation and nevus excision predominated in the Clean group (Table 4). The status of infection distribution did not differ significantly between the ARB and ASB groups (*P* = .97).

**Table 4:** Type, location, size, and coverage methods of wound.

	Group			Total	*P* value		
	ARB	ASB	Clean		ARB-ASB-clean	ARB- ASB	CLASS I–IV
Type of wound							
Pressure sore	42 (52.5%)	17 (23.6%)	–	59 (38.8%)			
Trauma	15 (18.8%)	23 (31.9%)	–	38 (25.0%)			
Surgical site infection	8 (10.1%)	20 (27.8%)		28 (18.5%)			
Burn	6 (7.5%)	9 (12.5%)	–	15 (9.9%)			
Chronic ulcer	6 (7.5%)	1 (1.4%)		7 (4.6%)			
Etc.	3 (3.8%)	2 (2.8%)	–	5 (3.3%)			
Soft tissue tumor excision	–	–	51 (100.0%)				
Location of wound							
Trunk	59 (73.8%)	42 (58.3%)	37 (72.5%)	138 (68%)			
Extremities	14 (17.5%)	25 (34.7%)	11 (21.6%)	50 (24.6%)			
Scalp	7 (8.7%)	5 (7%)	3 (5.9%)	15 (7.4%)			
Wound size (median, IQR)	29 (18,49)	25 (16,48)	18 (15,24)	25 (16,45)	0.002^∗^	0.47^†^	0.001^†^
Type of flap for reconstruction							
FC flap	28 (35%)	29 (40.3%)	44 (86.3%)	101 (49.8%)			
MC flap	49 (61.3%)	30 (41.7%)	7 (13.7%)	86 (42.4%)			
Free flap	3 (3.7%)	13 (18%)	0	16 (7.8%)			

ARB = antibiotic-resistant bacteria, ASB = antibiotic-sensitive bacteria, FC flap = Fasciocutaneous flap, IQR = interquartile range, MC flap = Myocutaenous flap.

∗ One-way ANOVA test.

† Independent *T* test.

Most wounds were located on the trunk (n = 138), followed by the extremities (n = 50) and scalp (n = 15) in all 3 groups. The distribution of wound location did not differ significantly among the 3 groups (*P* = .151).

The median wound size was largest in the ARB group (48 [range 4–450] cm^2^) followed by the ASB (41 [range 4–255) cm^2^] and Clean (19 [range 1–90] cm^2^) groups. Wound size differed among the 3 groups (*P* = .002). It was larger in the class IV wound group than the class I wound group (*P* = .001). However, it did not differ significantly between the ARB and ASB groups (*P* = .47).

In the ARB group, MRSA and *Acinetobacter* species were the most common pathogens, followed by *Pseudomonas* species. In the ASB group, *Enterococcus* species were most frequent (Table 5).

**Table 5:** Pathogens and healing time of wound.

	Group			Total	*P* value		
	ARB	ASB	Clean		ARB-ASB-clean	ARB-ASB	CLASS I–IV
Pathogens							
MRSA	32 (21.2%)						
Pseudomonas species	31 (20.5%)						
Acinetobacter species	32 (21.2%)						
Enterococcus species	31 (20.5%)	28 (36.3%)					
MR-CNS	15 (10.0%)						
VRE	5 (3.3%)						
CRE	5 (3.3%)						
No growth		26 (33.8%)					
MSSA		15 (19.5%)					
Enterobacteriaceae species		8 (10.4%)					
Healing time of wound (mean ± SD)	19.7 ± 12.0	17.9 ± 10.0	16.5 ± 4.8	30.4 ± 32.7	0.036^∗^	0.164^†^	0.01^†^

ARB = antibiotic-resistant bacteria, ASB = antibiotic-sensitive bacteria, CRE = Carbapenem-resistant Enterobacteriaceae, MR-CNS = Methicillin-resistant coagulase-negative Staphylococci, MRSA = Methicillin-resistant *Staphylococcus aureus*, MSSA = Methicillin-sensitive *Staphylococcus aureus*, SD = standard deviation, VRE = Vancomycin-resistant enterococci.

∗ One-way ANOVA test.

† Independent *T* test.

Myocutaneous flaps (n = 101) were most frequently used, followed by fasciocutaneous flaps (n = 86) and free flaps (n = 16). Appropriate antibiotics based on culture results were administered postoperatively (Table 4).

### 3.3 Healing time

**Figure 1:**
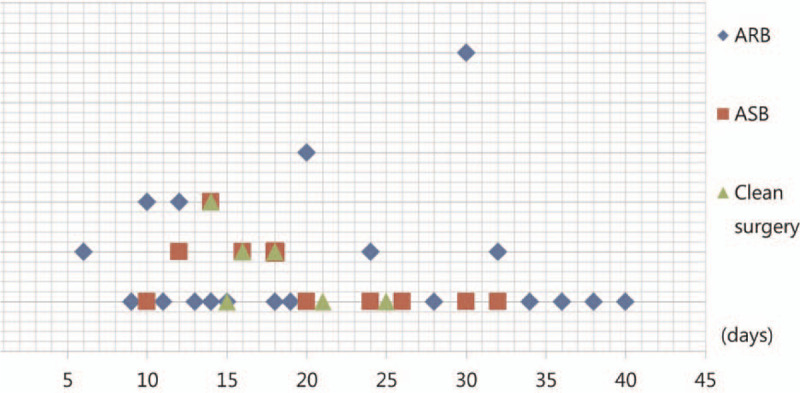
Comparison of the healing times of all patients included in the study. ARB = antibiotic-resistant bacteria group, ASB = antibiotic-sensitive bacteria group.

Average healing time was longer in the ARB (19.7 [range 7–44] days) and ASB (17.9 [range 2–36] days) groups than the Clean group (16.5 [range 7–28] days). Healing time differed among the 3 groups (*P* = .036). Class IV wounds took longer to heal than did class I wounds (*P* = .01). However, healing time did not differ significantly between the ARB and ASB groups (*P* = 0.164) (Table 4, Fig. 1).

### 3.4 Patient cases

#### 3.4.1 Case 1

**Figure 2:**
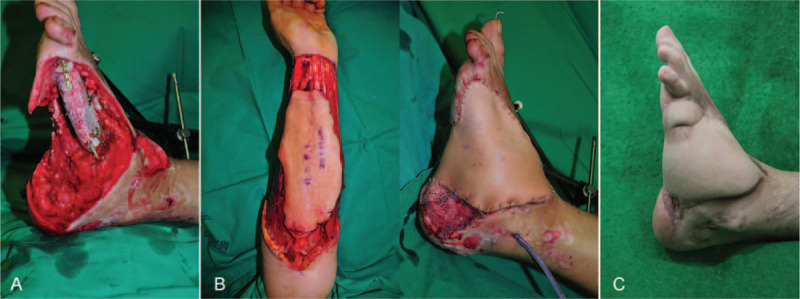
A, A complicated wound on left foot after a series of debridement. B, The wound was reconstructed with a free iliac bone and radial forearm free flap. C, At postoperative 1 year, the wound was healed well without any complications.

A 25-year-old male was in a tragic traffic accident, resulting in the loss of nearly one-third of his left foot and *Pseudomonas aeruginosa* was cultured from the wound (Fig. 2). Although the orthopedic department recommended amputation, we reconstructed the foot with a free iliac bone and radial forearm free flap. After 1 year, the infection was controlled and the patient had a normal gait without any other complications.

#### 3.4.2 Case 2

**Figure 3:**
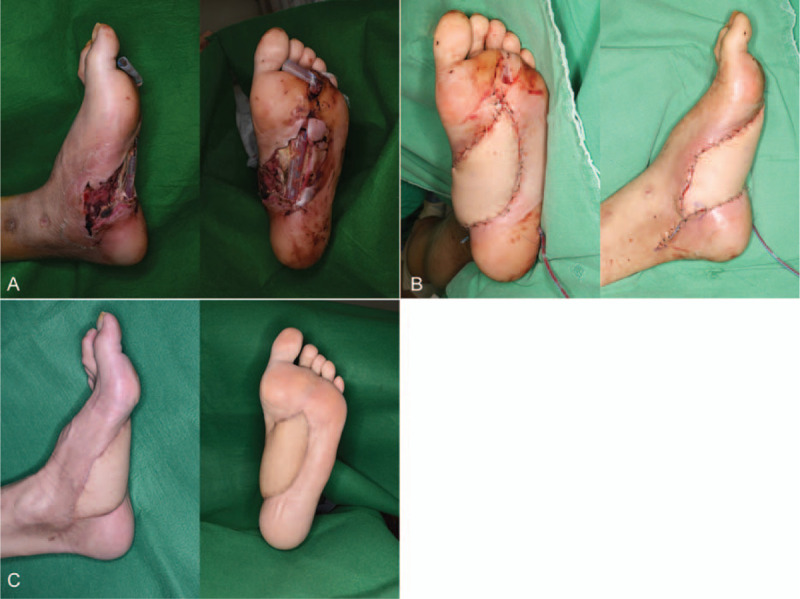
A, A complicated wound with the exposure of tendon, muscle, and bone on the left foot. B, The wound was covered with anterolateral thigh free flap. C, At postoperative 1 year follow-up, the wound healed well.

A 31-year-old male visited with a complicated wound on his left foot that spontaneously occurred due to diabetic mellitus type 1 under consultation of the Department of Orthopedic Surgery (Fig. 3). *Pseudomonas aeruginosa* was cultured from the wound. Orthopedic Department had planned to control the infection prior to surgery, but it was not improved. Soft tissue transfer with anterolateral thigh free flap was done. At postoperative 1 year follow-up, the wound healed well without any complications.

#### 3.4.3 Case 3

**Figure 4:**
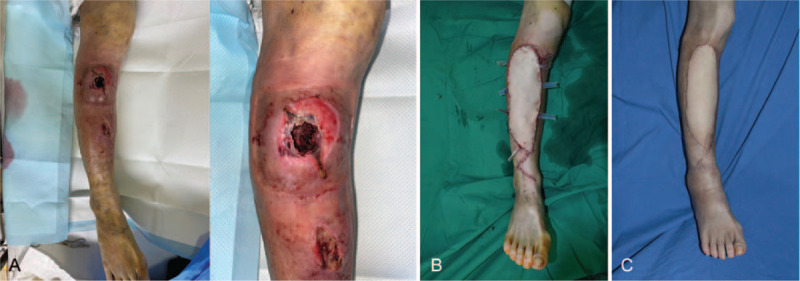
A, The wound with bone exposure was on the right pretibial area. B, The wound was covered with anterolateral thigh free flap. C, At postoperative 6 months. The result was satisfactory without any early and late complications.

A 58-year-old female was visited with a open wound that occurred after slipping down in the bathroom (Fig. 4). Methicillin-resistant *Staphylococcus aureus* was cultured in the wound. After 2 times of surgical debridement, microvascular surgery was performed within 3 weeks after injury. The result was satisfactory without any early and late complications.

## 4 Discussion

Antibiotic-resistant bacteria pose a great threat to the overall healthcare system, considering their increase in rate of infection and the difficulties in eradication.^[13]^ Particularly in wounds infected with antibiotic-resistant bacteria, an antibiotic-only approach would not be sufficient.^[14,15]^ Serial debridement and secondary healing of the wound often result in poor outcomes and recurrence in such cases. Some wounds can turn into chronic wounds, which prolong treatment and increase costs, and skin cancers may even occur.^[16]^

The patients were grouped based on tissue culture results. Conventionally, wounds with negative culture results or antibiotic-sensitive bacteria underwent surgical debridement without delay because the possibility of postoperative infection was estimated to be low with appropriate antibiotics. Meanwhile, soft tissue coverage of the infected wounds or any surgical approach in patients with antibiotic-resistant bacteria was delayed until culture results turned negative or infection signs resolved clinically.^[17]^ In this study, all 3 groups underwent surgical management of the wound as soon as conditions allowed for general anesthesia, which made direct comparison of the 3 groups with culture results possible.

**Figure 5:**
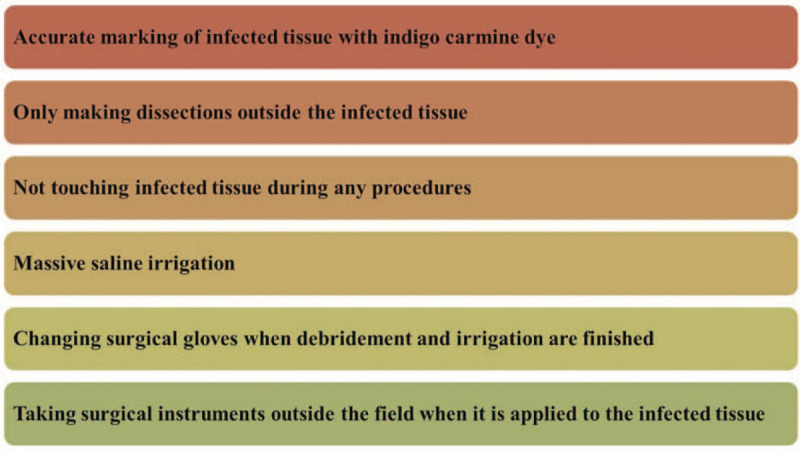
Specific guidelines in the operating room.

In our institution, the surgical guideline for infected wounds is quite simple, as suggested above. The most important principle is *en bloc* resection. Specific steps of *en bloc* resection are described in Figure 5. This refers to radical debridement of the infected tissue. The principle is similar to surgical approaches for skin cancer, which have safety margins to completely remove cancer cells. Although an adequate safety margin for *en bloc* resection has not been established, the goal of the procedure is to remove all unhealthy tissue, with normal, healthy, bleeding soft tissue or bone remaining after the procedure. We usually used a safety margin of approximately 2 cm.

In cases of osteomyelitis, there is mixed evidence on treatment protocols and some suggest a long duration of intravenous antibiotic injection, which lasts 4 to 6 weeks.^[18]^ After applying our treatment principles, we do not use intravenous antibiotics after removing the sutures, because the infected tissue, including bone with osteomyelitis, was removed. Bone debridement was also done radically, until fresh bleeding cancellous bone was seen.^[19]^ Bone was debrided until punctate bleeding on the cortex (paprika sign) and normal-looking marrow in cancellous bone.^[20]^ There were no complications regarding osteomyelitis.

Some patients, not included in the study, had undergone insufficient resection of infected tissue and experienced recurrent infections after multiple surgeries and extensive antibiotic use. A high recurrence rate after insufficient debridement is well established in the literature.

The optimal time for surgery for infected tissue has not been established. There are mixed reports about early and delayed soft tissue coverage.^[21–24]^ Delayed soft tissue coverage leads to prolonged hospitalization and antibiotic use and increases medical costs. Our approach for the timing of surgery was to attempt it as soon as possible. This is based on the following hypotheses: surgical eradication of the infection focus reduces bacterial load and decreases infection, thus boosting healing;^[25]^ when the pathogen is completely eliminated, no additional antibiotic is needed, which leads to decreased antibiotic use and fewer antibiotic-related complications; and the vascularized flap itself can help wound healing. With the surgical principles stated above, there was no need to wait for adequate infection control with antibiotics and serial debridement. When there were no complications, patients were discharged after the sutures were removed, usually not exceeding 2 weeks postsurgery. This practice has significantly reduced the period of hospitalization, infection rates, and duration of antibiotic use.

When a bacterial biofilm is established within a wound, the healing process is inhibited by the physical barrier that the biofilm creates for reepithelization^[26]^ and by the opsonization of bacteria.^[27]^ Also, the constant release of waste products induces a chronic inflammatory response in the surrounding tissue that interferes with wound healing. However, the principle can also be applied to a wound with a biofilm; the most important surgical step in treating any wound is to perform adequate debridement to remove all foreign material and unhealthy or nonviable tissue until the wound edges and base consist only of normal, healthy tissue.

Staphylococcus strain progresses in 2 forms: acute fulminant infection or chronic relapse-prone infection. Sandulescu et al^[28]^ introduce a method of predicting the pattern by scoring it with 3 characteristics—Staphylococcus aggressive score (SAS). Resistance to antibiotics acts as a factor, and resistance to specific antibiotics can increase the tendency to form biofilm and progress to chronic wounds.

Soft tissue coverage of the resultant soft tissue defect was achieved with vascularized flaps. This study deals with wounds with heavy bacterial loads requiring intensive care. In these cases, defects after debridement were usually large (mean wound size, 48 cm^2^; range, 4–450 cm^2^), regional flaps or free flaps were used, and underlying muscle or fascia were included to ensure vascularity. A decline in bacterial counts at the undersurface of musculocutaneous and fasciocutaneous flaps occurs within 24 hours and multiple reports suggest that vascularized flaps have positive effects on complicated bacterial infections.^[26,29]^ Some authors have demonstrated improvements in the dynamic properties of blood flow, oxygen concentration, leukocyte mobilization, and the intracellular killing activity of leukocytes within musculocutaneous flaps, so wound healing was better. Higher blood flow in these flaps results in increased antibiotic delivery, phagocytic activity, and bacterial clearance and enhanced leukocyte activity.^[27,30,31]^ Although skin flaps or perforator flaps were not used in the present study to ensure vascularity, there have been reports of the use of perforator flaps for infection control.^[32]^

There are several limitations to our study due to its retrospective design. First, the overview of all known clinical factors related to wound healing is limited. Second, further studies are needed to confirm our results and determine adequate resection margins. Third, complication rates when the wounds were inadequately debrided were not discussed. To our knowledge, this is the first report of a direct comparison of surgical outcomes between dirty wounds with antibiotic-resistant bacteria, nonresistant bacteria, and relatively clean surgeries. There are only anecdotal reports suggesting flap coverage has positive effects on such dirty wounds. Based on this report, surgeons can have confidence in treating antibiotic-resistant bacteria with proper techniques.

## 5 Conclusion

ARB-infected wounds are difficult to heal. Since there are few antibiotic options available and surgery is performed after controlling the infection, the treatment period is prolonged, which itself acts as a risk factor that causes the wound to worsen.

In our study, the difference in healing time was small when vascularized tissue transfer was done in ARB-infected wound compared with ASB-infected and clean wound. Compared with conventional ARB treatment, the duration of treatment and antibiotic use are reduced, which leads to a reduction in hospitalization and treatment costs. It is necessary to actively perform surgery using vascularized tissue for the infected wound of antibiotic-resistant bacteria.

## Author contributions

**Conceptualization:** Seonghwan Kim, In Suck Suh.

**Data curation:** Ju Ho Lee, Seong Eun Kim, Se Ho Shin.

**Methodology:** In Suck Suh.

**Project administration:** Hyeon Jo Kim.

**Resources:** Hyeon Jo Kim.

**Software:** Seong Joo Lee.

**Writing – original draft:** Seonghwan Kim, Ju Ho Lee.

**Writing – review & editing:** Jae Hyun Kim, In Suck Suh.
